# A Meta-Analysis of High Dose, Intermittent Vitamin D Supplementation among Older Adults

**DOI:** 10.1371/journal.pone.0115850

**Published:** 2015-01-20

**Authors:** Ya Ting Zheng, Qi Qi Cui, Yi Min Hong, Wei Guang Yao

**Affiliations:** College of Humanities and Management, Southern Medical University, Guangzhou, Guangdong Province, China; Garvan Institute of Medical Research, AUSTRALIA

## Abstract

**Background:**

The effects of intermittent, high dose vitamin D treatment in older adults have not been documented. We conducted a meta-analysis to provide a quantitative assessment of the efficiency of intermittent, high dose vitamin D treatment on falls, fractures, and mortality among older adults.

**Methods:**

Electronic databases were searched for randomized controlled trials (RCTs) on high dose, intermittent vitamin D supplementation among older adults. Two researchers independently screened the literature according to specified inclusive and exclusive criteria to extract the data. Meta-analysis was performed by using Review Manager 5.1.0 software.

**Results:**

Nine trials were included in this meta-analysis. High dose, intermittent vitamin D therapy did not decrease all-cause mortality among older adults. The risk ratio (95% CI) was 1.04 (0.91–1.17). No benefit was seen in fracture or fall prevention. The risk ratio for hip fractures (95% CI) was 1.17 (0.97–1.41) while for non-vertebral fractures (95% CI) it was 1.06 (0.91–1.22), and the risk ratio for falls (95% CI) was 1.02 (0.96–1.08). Results remained robust after sensitivity analysis.

**Conclusion:**

Supplementation of intermittent, high dose vitamin D may not be effective in preventing overall mortality, fractures, or falls among older adults. The route of administration of vitamin D supplements may well change the physiological effects.

## Introduction

Vitamin D plays a key role in human biology [1], and the beneficial effects of vitamin D supplementation have been documented. A large number of randomized controlled trials (RCTs) and meta-analyses have investigated the effects of vitamin D treatment on fractures and falls and suggest that vitamin D supplementation is effective in preventing fractures and falls among older adults [2–7]. Several meta-analyses of RCTs on the effect of vitamin D supplementation on total mortality have been published and found that when given together with calcium, vitamin D supplementation reduced total mortality but not when given alone [8–11].

In recent years, many researchers have focused on supplementing individuals with intermittent, high-dose vitamin D. A study of supplementation with 100,000 IU of oral vitamin D_3_ every three months demonstrated an increase in mean serum 25-hydroxy vitamin D, from 36.4 nmol/L at baseline to 124.0nmol/L at six months [12]. Another study compared the effects of vitamin D_3_ in a single dose of 500,000 IU with 50,000IU per month among older adults, concluding that large loading doses of vitamin D_3_ rapidly and safely normalized 25-hydroxy vitamin D levels in the frail older adult [13]. Finally, a RCT suggested that supplementation with cholecalciferol 100,000 IU every four months may prevent fractures without adverse effects in older adults living in the general community [14].

However, several RCTs using high dose, intermittent vitamin D reported an increase rather than a decrease, in the primary outcome of falls [15] and fractures [15,16]. This concern leaves the field with a challenge when considering the use of high dose of vitamin D supplementation among older adults. Thus, a meta-analysis that addresses this issue is clearly needed.

The objective of the current study was to perform a comprehensive systematic review and meta-analysis of RCTs to observe the effects of high dose, intermittent vitamin D on fall, fracture, and overall mortality prevention in older adults.

## Materials and Methods

### Search strategy

We performed a literature search for the purpose of identifying RCTs. We searched the electronic databases of Medline, Embase, and The Cochrane Central Register of Controlled Trials up to January 2013. The search strategy combined terms relating to study design (RCTs), intervention of vitamin D (vitamin D, vitamin D_2_, vitamin D_3_, ergocalciferol, cholecalciferol), and outcomes (falls, fractures, mortality). We also searched for any additional studies in the reference lists of recent reviewers of vitamin D treatment on older adults. Our searches were limited to human trials with no language restriction.

### Eligibility criteria

The search results were then screened on the basis of the following criteria: (1) RCTs referring to an annual high dose (greater than 100,000 IU) or intermittent dose (interval time longer than one month); (2) The participants of this study were individuals aged 65 years or over. Unstable conditions, such as stroke and Parkinson’s disease, were excluded; (3) The treatment group was restricted to high dose, intermittent vitamin D alone, or in combination with calcium. The control group had no treatment or calcium therapy. Studies of patients receiving active vitamin D were excluded from the present study; (4) The number of participants with one or more falls, fractures, and deaths was reported separately for the vitamin D treatment group and the control group; (5) Review articles, commentaries, letters, observational studies were excluded.

### Data extraction and quality assessment

Two researchers independently and in duplicate abstracted data using a standardized form. Data collected from studies included first author, publishing year, sample size, duration, dwelling, intervention, serum 25-hydroxy vitamin D levels at baseline, and primary results (the number of participants who suffered at least one fall, fracture, or death). Total number of falls and fractures were not used in the present study, because individuals with recurrent falls and fractures may have other significant risk factors, which may exaggerate the estimated risk. Quality assessment was performed by two independent researchers using the Cochrane Collaboration’s tool [17].

### Data synthesis and analysis

The primary outcome was the number of participants who died during follow-up. Secondary outcomes were the number of participants who suffered hip fractures, non-vertebrae fractures, and falls. Mantel-Haenszel method was used to calculate risk ratios (RRs) and their 95% confidence intervals (CI). The I^2^ statistic was used to assess the presence of heterogeneity, I^2^ range from 0% to 100%. An I^2^ statistic greater than 50% suggested moderate heterogeneity [18], and a random effects model was used. A fixed-effects model was used for I^2^ statistic less than 50%, which showed that heterogeneity could be neglected [18]. We used Begg test and Egger test to evaluate the publication bias regarding the RR of mortality [19,20]. A p value less than 0.05 was considered to be statistically significant.

### Sensitivity analysis and subgroup analysis

A sensitivity analysis was conducted by using the trim and fill method. For the overall results of mortality and fractures, we performed a sensitivity analysis by omitting one study at a time and then repeating the analysis. For the falls outcome, sensitivity analysis was conducted by excluding trials which did not have an explicit fall definition.

To explore the causes of inconsistency and sub-group treatment interactions, we conducted sub-group analyses according to type of residence (institutionalized vs. community dwelling), type of vitamin D (ergocalciferol vs. cholecalciferol), route of administration (intramuscular vs. oral), baseline level of 25-hydroxy vitamin D (≤ 50 nmol/liter vs. > 50 nmol/liter), and duration of vitamin D treatment (< 3 years vs. ≥ 3 years).

## Results

### Search Results

**Figure 1:**
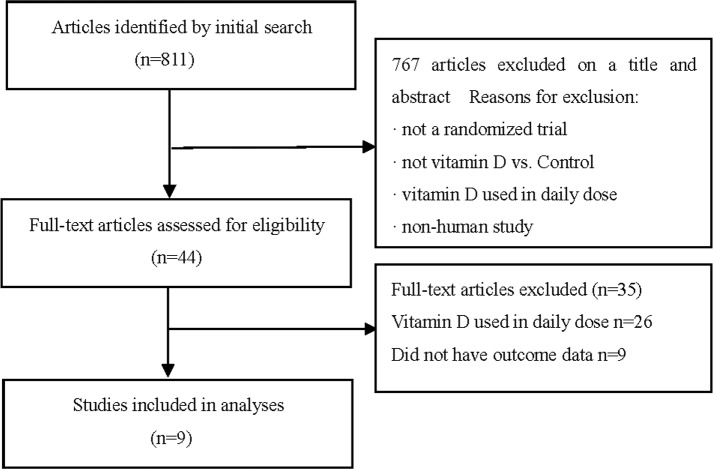
Study flow diagram.

Eight hundred and eleven potentially relevant publications were found after an initial independent search of the electronic database. After scrutinizing the titles and abstracts, 767 articles were excluded according to the inclusion criteria. Among the remaining forty-four articles, twenty-six were excluded because vitamin D was used daily, nine were excluded because they did not provide acquired data. Subsequently, a total of nine RCTs were included in the final analysis [14–16, 21–26]. The details of study selection flow were explicitly described in Fig. 1.

### Study Characteristics

The main characteristics of the included studies are shown in Table 1. Nine trials containing 22,012 patients (10,950 in the vitamin D group and 11,062 in control group) were included in the present study. Mortality data was available in seven trials [14–16, 21, 22, 24, 25], four trials had hip fracture data [14, 16, 24, 25], and five trials had non-vertebral fracture data [14–16, 24, 25]. Eight trials reported fall data but only three were included in the primary analysis for their definition of falls and how they were assessed [15, 23, 26]. Duration of follow-up ranged from six months to five years. The trials were published from 2003 to 2012. Mean age of participants ranged from seventy-seven to eighty-five years. Vitamin D_2_ was used in six studies [16, 21–25] and vitamin D_3_ in the remaining three studies. Six trials [14, 15, 21, 24–26] used oral vitamin D while intramuscular injection was used in the remaining three trials. Calcium supplementation was used in two trials [22, 26]. All trials reported the baseline vitamin D status of participants based on serum 25-hydroxy vitamin D levels. Participants in six trials [14–16, 24–26] had baseline 25-hydroxy vitamin D levels at or above vitamin D adequacy (20 ng/ml or 50 nmol/L). Participants in the remaining three trials had baseline 25-hydroxy vitamin D levels in a range considered to be vitamin D deficient (< 20 ng/ml). Two had a high risk bias [22, 24], and the other seven studies had a low risk bias (Table 2).

**Table 1:** Characteristics of studies included in primary analysis.

**Study**	**Population characteristics**	**Treatment groups**	**Number of participants**	**Mean age (years)**	**Pre/post 25(OH)D (nmol/l;mean)**	**Follow up (moths)**	**Outcomes**
Latham 2003 [21]	Recruited from geriatric rehabilitation center, institutionalized	Oral vit D_2_ 300000 IU once	108	80	37 to 60 at 3 months	6	Mortality
		Placebo	114	79	48 to 48 at 3 months		
Trivedi 2003 [14]	Elderly man and woman, community dwelling	Oral vit D_3_ 100000 IU every 4 months	1027	75	74 at 48 months	60	Mortality, fracture
		Placebo	1011	75	53 at 48 months		
Harwood 2004 [22]	Elderly women after hip fracture, community dwelling	Vit D_2_ 300,000 IU/im/once	30	80	28 to 41 at 12 months	12	Mortality
		Vit D_2_ 300,000 IU/im/once + 1,000 mg calcium	25	81	30 to 48 at 12 months		
		No treatment	35	81	30 to 27 at 12 months		
Dhesi 2004 [23]	Ambulatory elderly with a history of fall institutionalized	Vit D_2_600000IU/im/once	62	77	27 to 44 at 6 months	6	Fall
		Placebo	61	77	25 to 31 at 6 months		
Law 2006 [24]	Recruited from residential care home institutionalized	Oral vit D_2_ 100000 IU every 3 months	1762	85	59 to 77 at 3 months	10	Mortality Fracture
		No treatment	1955	85	NA		
Lyons 2007 [25]	Nursing home residents institutionalized	Oral vit D_2_ 100000 IU every 4 months	1725	84	80/NA	36	Mortality fracture
		Placebo	1715	84	54/NA		
Smith 2007 [16]	Elderly man and woman, community dwelling	Vit D_2_ 300,000 IU/im/year	4727	79	56.5 to 68 at 4 months	36	Mortality fracture
		Placebo	4713	79	NA		
Sanders 2010 [15]	Ambulatory elderly women at risk for fractures, community dwelling	Oral vit D_3_ 500000 IU annually for 3–5 yerrs	1131	77	53/NA	36–60	Mortality fracture
		Placebo	1125	77	45/NA		fall
Glendenning 2012 [26]	Older women, community dwelling	Oral vit D_3_150000 IU every 3 months + calcium 1300 daily	353	77	65 to 75 at 9 months	9	Fall
		Calcium 1300 daily	333	77	66 to 60 at 9 months		

**Table 2:** Quality assessment of the included studies.

**Study[ref]**	**Random sequence generation**	**Allocation concealment**	**Blinding of participant and personnel**	**Blinding of outcome assessment**	**Incomplete outcome data addressed**	**Nonelective reporting**	**Other bias**
Latham 2003	L	L	L	L	L	L	L
Trivedi 2003	L	L	L	L	L	L	L
Harwood 2004	L	L	H	H	L	L	H
Dhesi 2004	L	L	L	L	L	L	L
Law 2006	L	H	H	H	L	L	U
Lyons 2007	L	L	L	L	L	L	L
Smith 2007	L	L	L	L	L	L	L
Sanders 2010	L	L	L	L	L	L	L
Glendenning 2012	L	L	L	L	L	L	L

L, low risk; H, high risk; U, unclear

### Meta-analysis of high dose, intermittent vitamin D treatment on overall mortality

**Figure 2:**
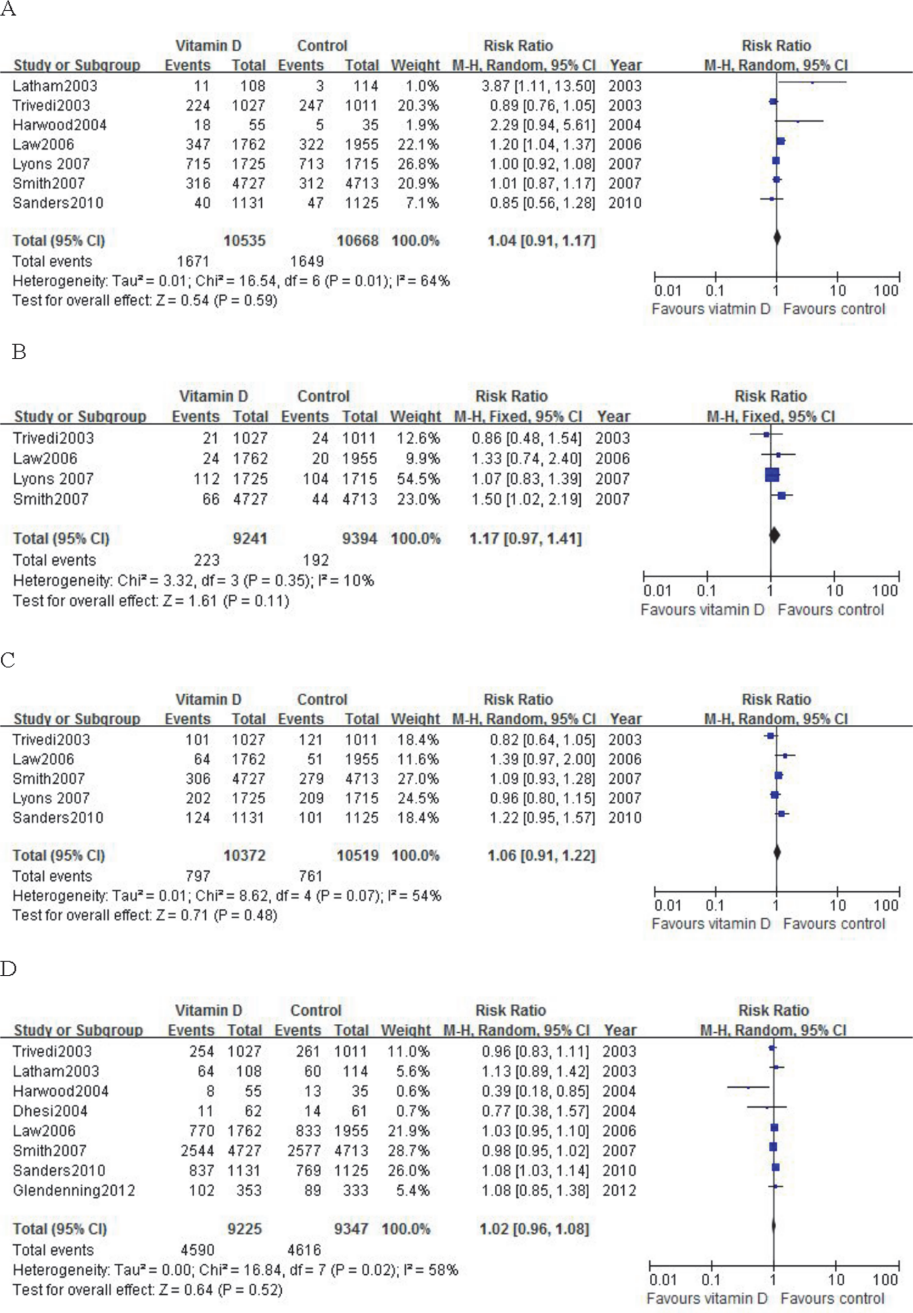
Meta analysis of overall mortality (A), hip fracture (B), non-vertebral fracture (C), fall (D) in participants treatment with high dose, intermittent vitamin D compare with control.

The risk ratio (95% CI) of mortality for patients treated with high dose, intermittent vitamin D compared with control was 1.04 (0.91–1.17), which was not statistically significant (p = 0.59). Heterogeneity was noted for this outcome (I^2^ = 64%) (Fig. 2A). A total of 1671 of 10,535 participants (15.9%) randomized to the vitamin D group and 1649 of 10,668 participants (15.5%) randomized to the placebo or no intervention group died. Results remained robust after sensitivity analysis.

Sub-group analysis (Table 3) showed no appreciable change in risk ratio according to gender, participant residency, type of vitamin D, route of administration, baseline 25-hydroxy vitamin D, or trial duration.

**Table 3:** Subgroup analyses of high-dose, intermittent vitamin D treatment on mortality.

**Subgroup**	**Studies,n**	**Participants, n**		**Death, n**		**Risk Ratio**	**P value**	**I^2^value**
		Treatment	Control	Treatment	Control	(95% CI)		
Participant dwelling								
Institutionalized	3	3595	3784	1073	1038	1.13 (0.90, 1.41)	0.29	79%
Community-dwelling	4	6940	6884	598	611	0.96 (0.87, 1.07)	0.48	43%
Gender								
Women only	3	1512	1483	83	79	1.06 (0.66, 1.68)	0.82	50%
Men only	1	1019	1018	199	220	0.90 (0.76, 1.07)	0.25	0%
Type of vitamin D								
Ergocalciferol	5	8377	8532	1407	1355	1.10 (0.95, 1.29)	0.21	68%
Cholecalciferol	2	2158	2136	264	294	0.89 (0.76, 1.03)	0.11	0%
Administration route								
Intramuscular	2	4682	4748	334	317	1.03 (0.89, 1.20)	0.66	68%
Oral	5	5753	5920	1337	1332	1.02 (0.96, 1.09)	0.46	70%
Baseline 25-hydroxy vitamin D								
≤ 50 nmol/liter	2	163	149	29	8	2.80 (1.36, 5.78)	0.005	0%
> 50 nmol/liter	5	10372	10519	1642	1641	1.01 (0.91, 1.12)	0.83	55%
Duration of vitamin D treatment								
≥ 3 years	4	8610	8564	1295	1319	0.98 (0.91, 1.04)	0.44	0%
< 3 years	3	1925	2104	376	330	1.80 (0.90, 3.60)	0.09	62%

For I^2^ ≥50%, random effects model was used; for I^2^ <50%, fixed-effects model was used

### Meta-analysis of high dose, intermittent vitamin D treatment on fractures

The risk ratio (95% CI) of hip fracture for patients treated with high dose, intermittent vitamin D compared with control was 1.17 (0.97–1.41), which was not statistically significant (p = 0.11). Heterogeneity was insignificant for this outcome (I^2^ = 10%) (Fig. 2B). The risk ratio (95% CI) of non-vertebral fracture for patients treated with high dose, intermittent vitamin D compared with control was 1.06 (0.91–1.22), which was not statistically significant (p = 0.48). Heterogeneity was noted for this outcome (I^2^ = 54%) (Fig. 2C). Results were robust in the sensitivity analysis by excluding individual trials one by one.

### Meta-analysis of high dose, intermittent vitamin D treatment on falls

The risk ratio (95% CI) of falls for patients treated with high dose, intermittent vitamin D compared with control was 1.02 (0.96–1.08), which was not statistically significant (p = 0.52). Heterogeneity was significant for this outcome (I^2^ = 58%) (Fig. 2D). In sensitivity analysis of vitamin D treatment on falls, we excluded the trials where the definition of falls was not explicitly given. The risk ratio (95% CI) of falls for patients treated with high dose, intermittent vitamin D compared with control after sensitivity analysis was 1.08 (1.02–1.14), which was statistically significant (p = 0.006). Heterogeneity was insignificant for this outcome (I^2^ = 0%).

### Publication bias

No evidence of publication bias was detected for the RR of mortality in the present study by either Begg or Egger’s test (Begg’s test, p = 0.764; Egger’s test, p = 0.295).

## Discussion

We aimed to bring together the results of all eligible RCTs to gauge the effect of high dose, intermittent vitamin D supplementation among older adults concerning mortality, and fracture and fall prevention. Our results demonstrated that high dose, intermittent vitamin D supplementation was ineffective in preventing mortality. It was fairly consistent across sub-groups defined by participant residency, type of vitamin D, route of administration, baseline 25-hydroxy vitamin D, and trial duration. In addition, no benefit was seen in fracture and fall prevention.

Several previous meta-analyses suggested that oral vitamin D treatment reduced the risk of fractures and falls among older individuals [4–7], and further meta-analyses suggested that vitamin D was effective in reducing mortality [8–11]. Most studies included in these meta-analyses [4–11] were referring to vitamin D intake in the form of a daily tablet. Different from previous studies, the present study only involved trials using high dose (a single dose larger than 100,000 IU) or intermittent vitamin D (interval time longer than one month). The results contradicted previous findings.

A fall endpoint study of oral cholecalciferol 1000 IU plus calcium 1000 mg daily for one year in 302 community-dwelling older women living in Australia demonstrated a 19% reduction of first fall events [27]. Another RCT conducted by Flick et al [28] including 625 older people residing in sixty hostels and eighty-nine nursing homes across Australia demonstrated that ergocalciferol administered 10,000 IU once weekly and then 1,000 IU daily was associated with a significant reduced risk of falls and fractures. The results of these two trials indicated that high daily dose vitamin D was effective in preventing falls and fractures. In contrast, our results indicated that high dose, intermittent vitamin D was not effective in preventing falls and fractures. This may suggest that the dosing regimen, rather than the total dose, might determine the outcome [29]. Annual single-dose or intermittent, high dose vitamin D may not provide adequate concentrations in the blood over a whole year. Thus, negative outcomes may occur due to failure to maintain serum vitamin D over time, as with monthly, quarterly, or yearly bolus vitamin D dosing [30].

A systematic review concluded that cholecalciferol significantly decreased mortality while the effect of ergocalciferol may be neutral or even detrimental [10]. One included trial in the present study by Trivedi et al [14] suggested that four monthly supplementations with 100,000 IU cholecalciferol were beneficial for fractures and falls, and may prevent overall mortality in elderly living in the general community. However, four other studies demonstrated that ergocalciferol 300,000 IU once yearly or 100,000 every four months was not effective in preventing mortality and fractures. These five trials used the same total annual dose of vitamin D, but outcomes were entirely different. It may indicate that annual high doses of cholecalciferol may be more beneficial than ergocalciferol with certain time intervals.

The deleterious effect of high dose, intermittent vitamin D was observed in two studies [15,16]. Sanders et al [15] concluded that annual oral administration of 500,000 IU cholecalciferol resulted in an increased risk of falls and fractures; Smith et al [16] demonstrated that vitamin D was associated with a significantly increased risk of hip fractures. In the present study, the primary analysis of high dose, intermittent vitamin D treatment on falls found a significantly increased risk. The mechanism of the deleterious effect of supplemental intermittent, high-dose of vitamin D remains uncertain. The effect of vitamin D on muscle tissue is thought to occur through specific vitamin D receptors, and vitamin D receptor expression is decreased in older adults [31]. When vitamin D is given intermittently or daily, it has positive effects on muscle function, but when a very high dose of vitamin D is given, there is a negative effect on muscle function due to a sudden increase in vitamin D receptor occupancy [29]. Vitamin D receptors also exist in the central nervous system [32], so a deleterious effect on falls is also possible [29]. It has been recently reported that mega-doses of vitamin D were associated with transient increases in serum 25-hydroxy vitamin D level and in bone turnover markers [33]. This might explain the transient increases in fracture rate observed by Sanders et al [15].

Our review has several limitations. First, most of the participants in the present study were older women. The effects of intermittent, high-dose of vitamin D in younger, healthy persons and males are still inconclusive. Second, ascertainment of falls and fractures varied between studies, which likely produced inaccuracies in outcome reporting. Third, the falls analysis was heavily influenced by two studies [15, 26], both of which used participants with a relatively high baseline 25-hydroxy vitamin D concentration (50 nmol/L). This means that the fall risk calculated here may not be applicable to persons with a low 25-hydroxy vitamin D concentration (50 nmol/L). It is possible that high dose, intermittent supplementation of vitamin D may be detrimental to those with a sufficient vitamin D status, but not to those with a poor vitamin D status. Fourth, though our results indicated that intermittent, high-dose of vitamin D was ineffective in preventing fractures, falls, and mortality, the threshold of high dose, interval time, and dosing regimen still have not been documented. It is still unknown what the optimal dosing strategy is.

In conclusion, high-dose, intermittent vitamin D may not have a beneficial effect on general health among older adults as has been previously described with a normal daily dose of vitamin D. The effect was likely neutral. For those with high levels of vitamin D (25-hydroxy vitamin D levels > 50 nmol/L) to begin with, there may be a deleterious effect on falls. However, randomized controlled trials with physical outcomes as primary end points, using a number of different regimens are still needed to definitively resolve this challenging issue.

## Supporting Information

S1 PRISMA Checklist. (DOC)
